# The Role of the BH4 Cofactor in Nitric Oxide Synthase Activity and Cancer Progression: Two Sides of the Same Coin

**DOI:** 10.3390/ijms22179546

**Published:** 2021-09-02

**Authors:** Diego Assis Gonçalves, Miriam Galvonas Jasiulionis, Fabiana Henriques Machado de Melo

**Affiliations:** 1 Micro-Imuno-Parasitology Department, Universidade Federal de São Paulo, São Paulo 04023-062, Brazil; diegoassisg@gmail.com; 2 Department of Parasitology, Microbiology and Immunology, Federal University of Juiz de Fora, Juiz de Fora 36036-900, Brazil; 3 Pharmacology Department, Universidade Federal de São Paulo, São Paulo 04023-062, Brazil; mjasiulionis@gmail.com; 4 Department of Pharmacology, Institute of Biomedical Science, University of São Paulo, São Paulo 05508-000, Brazil; 5 Institute of Medical Assistance to Public Servants of the State (IAMSPE), São Paulo 04039-000, Brazil

**Keywords:** cancer, nitric oxide synthase, tetrahydrobiopterin, sepiapterin

## Abstract

Cancer development is associated with abnormal proliferation, genetic instability, cell death resistance, metabolic reprogramming, immunity evasion, and metastasis. These alterations are triggered by genetic and epigenetic alterations in genes that control cell homeostasis. Increased reactive oxygen and nitrogen species (ROS, RNS) induced by different enzymes and reactions with distinct molecules contribute to malignant transformation and tumor progression by modifying DNA, proteins, and lipids, altering their activities. Nitric oxide synthase plays a central role in oncogenic signaling modulation and redox landscape. Overexpression of the three NOS isoforms has been found in innumerous types of cancer contributing to tumor growth and development. Although the main function of NOS is the production of nitric oxide (NO), it can be a source of ROS in some pathological conditions. Decreased tetrahydrobiopterin (BH4) cofactor availability is involved in NOS dysfunction, leading to ROS production and reduced levels of NO. The regulation of NOSs by BH4 in cancer is controversial since BH4 has been reported as a pro-tumoral or an antitumoral molecule. Therefore, in this review, the role of BH4 in the control of NOS activity and its involvement in the capabilities acquired along tumor progression of different cancers was described.

## 1. Background

Cancers are a set of diseases characterized by genetic instability, abnormal cell proliferation, cell death resistance, metabolic reprogramming, angiogenesis, metastasis capability, and immune response evasion caused by genetic and epigenetic alterations in oncogenes and tumor suppressor genes [1]. These alterations contribute to malignant transformation and tumor development with the consequent acquisition of an increasingly aggressive phenotype through dysregulation of signaling pathways that maintain cell homeostasis. Despite the advancement of medical technology and the development of new target therapies [2,3], the incidence of different cancers is rising worldwide and will continue to increase over the course of this century according to the International Agency for the Research on Cancer (IARC) GLOBOCAN database within the Global Cancer Observatory (COSMIC v94, released 28-MAY-21). This scenario is mainly attributed to high life expectancy, the obesity epidemic, increased ultraviolet (UV) radiation exposure, and infectious pathogens classified as human carcinogens [4,5,6].

Among other cause effects, tobacco, obesity, and related metabolic syndromes, infections, and UV are associated with inflammation, loss of redox homeostasis, and oxidative stress, contributing to cancer development [7,8,9,10]. A pro-oxidant milieu alters redox signaling pathways improving the acquisition of cancer-related hallmarks. Moreover, nitric oxide (NO) and reactive nitrogen species (RNS) metabolism are also altered in inflammation [11]. High reactive oxygen species (ROS) levels found in tumors are caused by different mechanisms, including increased NADPH oxidase expression and activity, mitochondrial damage, endoplasmic reticulum stress, and nitric oxide synthase dysfunction (NOS) [12,13,14,15,16].

## 2. Nitric Oxide Synthase

**Figure 1:**
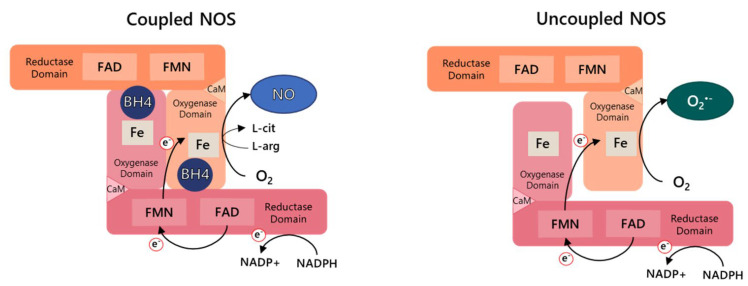
Nitric oxide synthase (NOS) activity. NOSs are homodimers and each monomer contains a reductase domain and an oxygenase domain connected by a calmodulin-binding peptide linker (CaM) (for eNOS and nNOS). In the normal NOS coupled state, electron (e^−^) flow through the nicotinamide adenine dinucleotide phosphate (NADPH) and flavin domains, adenine dinucleotide (FAD), and flavin mononucleotide (FMN), from the reductase domain of one monomer to the iron of the heme group (Fe) localized in oxidase domain of the other monomer. (6R)-5,6,7,8-tetrahydrobiopterin (BH4) binding to the heme active site at the interface between the two monomers increased L-arg (L-arginine) substrate interaction and dimer stabilization, producing nitric oxide (NO) and L-cit (L-citrulline). In the uncoupled state caused by the absence of BH4, the electron transfer and the reduction of oxygen (O_2_) are uncoupled from L-arginine oxidation resulting in the generation of superoxide anion (O_2_^•^^−^).

Three NOS (EC 1.14.13.39) isoforms have been described in mammalian cells. NOS isoforms, named neuronal NOS (*NOS1*), endothelial NOS (*NOS3*), and inducible NOS (*NOS2*), are encoded by different genes localized in distinct chromosomes. All three isoforms are homodimeric and each monomer contains a C-terminal reductase domain and an N-terminal oxygenase domain connected by a calmodulin-binding peptide linker. The binding sites for the cofactors flavin mononucleotide (FMN), flavin adenine dinucleotide (FAD), and nicotinamide adenine dinucleotide phosphate (NADPH) are found in the reductase domain, which shares homology with cytochrome P450 (CYT P450) reductases while the oxygenase domain contains binding sites for heme, the cofactor (6R)-5,6,7,8-tetrahydrobiopterin (BH4) and the substrate L-arginine. The electron transfer occurs from the NADPH cofactor into the oxygenase domain, where the L-arginine hydroxylation and subsequently NO formation takes place. Stable dimerization between the two NOS monomers is required for efficient NOS activity, as electrons flow from the reductase domain of one monomer to the oxidase domain localized in the other monomer (Figure 1) [17,18]. NOSs activity is regulated by integrating mechanisms, including post-translational modifications, protein–protein interactions, and substrate and cofactor availability, being BH4 concentration determinant [19,20,21].

NO is a pleiotropic gaseous molecule with a reactive free radical activity. NO regulates innumerous physiological processes, including vascular (blood flow, angiogenesis, platelet aggregation) and neurological functions (nervous system development and neurotransmission) [21]. Decreased NO concentration is associated with distinct diseases, including cardiovascular diseases, diabetes, autism, and cancer [22,23,24].

Different studies have shown that NO can promote or attenuate cancer progression through the regulation of diverse signaling pathways. Effects of NO on neoplasia depend on its concentration and exposure duration, NOS localization, NO-induced post-translational modifications of proteins, and cancer type and stage. The role of NO on cancer development is also associated with its source, tumor, or stromal cells (cancer-associated fibroblasts, endothelial or immune cells) [25,26]. At relatively high concentrations, NO induces cytotoxic and genotoxic events such as protein activity inhibition, mitochondria dysfunction, DNA and protein damage, and cell death while in low doses can trigger cell proliferation and angiogenesis. Many studies have reported the dual effect of NO in cancer cells [21,26]. In melanoma cells, increased NO concentration causes growth arrest and apoptosis [13,27]. Evodiamine treatment induced p38 and NF-κB activation, which in turn triggers NO-derived iNOS, increasing apoptosis in a p53-dependent manner [28]. Moreover, NO stabilizes p53 by phosphorylation, stimulating apoptosis in different cancers [29,30]. On the other hand, NO elicits HIF-1α accumulation in the nucleus of different tumor cells, enhancing gene expression of hypoxia-target genes, which in turn contributes to cancer proliferation, angiogenesis, and metastasis [31,32,33]. Furthermore, Newton et al. found that iNOS inhibition improved immunotherapy in combination with radiotherapy in solid tumors by modulating the tumor immune microenvironment. Decreased NO levels induced CD8^+^ effector cells activation and intratumoral infiltration, attenuating tumor growth [34].

Dysfunctional NOS is a source of superoxide anion (O_2_^•−^), which is associated with the development of cardiovascular diseases, diabetes, neurodegenerative disorders, and more recently, its role in cancer has been reported [13,35,36,37]. The role of ROS in malignant transformation and tumor progression depends on its concentration and cancer stage, contributing to carcinogenesis or tumor cell death [13,16,33].

Although increased expression of the three NOS isoforms in different cancers has been related in innumerous studies, not all the studies describe NOS activity [13,38,39,40,41]. High NOS expression is associated with increased cell proliferation, metastasis, and chemotherapy resistance, in some cases being associated with poor prognosis [26,40,42]. Therefore, the knowledge of NOS activity is very important to understand its real role in oncogenesis and to develop an efficient therapeutic strategy.

## 3. Tetrahydrobiopterin

BH4 plays an essential function as a cofactor of a set of metabolic enzymes, including NOS, four aromatic amino acid hydroxylases, alkylglycerol monooxygenase (AGMO), and the brain-specific tryptophan hydroxylase isomer TPH2. BH4 is a pteridine bicyclic molecule comprising a pyrimidine ring fused to a pyrazine ring. Biopterin’s redox activity is conferred by their heteroatomic rings that can be oxidized (biopterin), partially reduced (dihydrobiopterin, BH2), and fully reduced (BH4) [19]. BH4 also has the ability of scavenging ROS by preventing polyunsaturated fatty acyl (PUFA) chains depletion and lipid peroxidation [43] acting as an anti-oxidant molecule [44]. As BH4 is easily oxidized, an increase in ROS and RNS like peroxynitrite leads to BH4 depletion.

**Figure 2:**
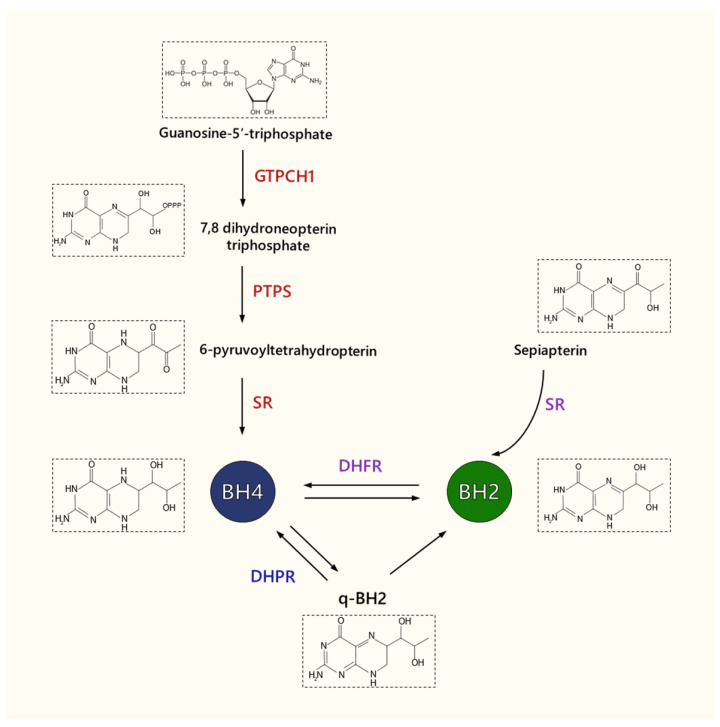
Tetrahydrobiopterin (BH4) biosynthesis pathway. *De novo* (red): BH4 is produced from guanosine-5′-triphosphate (GTP) by enzymes GTP cyclohydrolase 1 (GTPCH1), 6-pyruvoyl-tetrahydrobiopterin synthase (PTPS), and sepiapterin reductase (SR). Salvage (purple): Sepiapterin reduction into dihydrobiopterin (BH2) by SR, and BH2 conversion into BH4 by dihydrofolate reductase (DHFR). Recycling (blue): quinoid-BH2 reduction into BH4 by dihydropteridine reductase (DHPR).

Intracellular BH4 amount is tightly regulated by *de novo*, salvage, and recycle synthesis pathways (Figure 2) [45]. In *de novo* BH4 biosynthesis, BH4 is synthesized from GTP in a three-step reaction being GTPCH1 (GTP cyclohydrolase 1) (EC 3.5.4.16), the first and rate-limiting enzyme, followed by PTPS (6-pyruvoyl-tetrahydrobiopterin synthase) (EC 4.6.1.10) and SR (sepiapterin reductase) (EC 1.1.1.153) [46]. In the salvage pathway, SR catalyzes the reduction of sepiapterin into BH2 which is then converted into BH4 by DHFR (dihydrofolate reductase) (EC 1.5.1.3). In the recycling, quinoid-BH2 is reduced by DHPR (dihydropteridine reductase) (EC 1.5.1.34) into BH4. Other enzymes share with SR the same substrates, namely, AR (aldose reductase) and CR (carbonyl reductase). Additionally, BH4 can also be synthesized by AKR1C3 (3*α*-hydroxysteroid dehydrogenase type 3) (EC 1.1.1.357) and AR [47]. GTPCH1 activity is modulated in transcriptional and post-transcriptional levels and by interaction with the proteins GFRP (GTP cyclohydrolase I feedback regulator protein) and caveolin-1 [46,48,49]. GTPCH1 expression is triggered by pro-inflammatory cytokines, ROS, and NRF2 transcription factor in different cell lines [50,51].

BH4 binding to the heme active site at the interface between the two monomers is indispensable for NO synthesis through increased L-arginine substrate interaction and dimer stabilization. Decreased intracellular BH4 concentration promotes NOS destabilization and the reduction of NO production. This dysfunctional state of NOS is referred to as uncoupling because the oxidation of NADPH and the reduction of oxygen are uncoupled from arginine hydroxylation and NO formation. However, the electron transfer from NADPH through the flavin domains to molecular oxygen is not inhibited, resulting in the generation of O_2_^•−^ and hydrogen peroxide (H_2_O_2_) (Figure 1) [47]. Although BH2 can bind to the active site of NOS with the same affinity of BH4, it has no cofactor activity, competing with and displacing BH4 from the oxygenase domain. Therefore, the BH4/BH2 ratio also determines NOS function.

## 4. Tetrahydrobiopterin and Cancer

Different molecules have been described to contribute positively or negatively to tumor progression, which is associated with several factors, including the type of neoplasia and the stage of tumor development, reflecting the cross-talking between altered oncogenic signaling pathways and the interaction with other molecules. The compartmentalization within the cell and the location in the cancer microenvironment in addition to the expression and/or concentration of such factors also cooperate with its role.

As discussed below, the involvement of tetrahydrobiopterin as a pro- or anti-tumoral molecule has been demonstrated in different biological processes, including tumor microenvironmental reprogramming, cell growth, metabolism, and metastasis, contributing or impairing cancer development (Table 1).

### 4.1. Cell Growth

Proliferation is a biological process controlled by innumerous signaling pathways triggered by ligand-receptor interaction, which in turn, among other processes, regulates cell cycle progression, nutrition status, and genome integrity. In cancer, uncontrolled cell growth is associated with genetic and epigenetic alterations of key genes that maintain cell homeostasis and also by cell metabolism dysregulation [1,69].

As discussed, NOS coupling does not only depend on the total amount of BH4. The BH4/BH2 ratio and the stoichiometric balance between BH4 and NOS have been shown to determine the functional state of the enzyme [47,70]. In this context, some studies have shown that the increase in BH4/BH2 ratio, due to BH4 or precursor sepiapterin supplementation, impaired or improved cancer development, by regulating tumor growth by different mechanisms.

Rabender et al. observed decreased BH4/BH2 ratio in human breast cancer cells grown in culture, as xenografts, or in a spontaneous mouse breast cancer model (MMTVneu). Besides that, a low BH4/BH2 ratio was found in human colorectal carcinoma biopsies compared with the adjacent normal colon tissue. Treatment with L-sepiapterin partially reduced O_2_^•−^ and increased cGMP levels in MCF-7 and MDA-231 cancer breast cells, which suggested NOS recoupling. Moreover, L-sepiapterin supplementation of MCF-7 cells induced cGMP-dependent protein kinase (PKG) activation, which in turn promoted β-catenin expression and decreased TCF4 promoter activity [35]. The pro-inflammatory transcription factor NF-κB is associated with carcinogenesis since it induces the expression of different oncogenes [71]. The activity of NF-κB is both positively and negatively modulated by ROS/RNS since Tyr181 nitration of IκBa, an NF-κB inhibitor, triggers NF-κB activation. On the other hand, CYs38 S-nitrosylation of the NF-κB p65 subunit induces its inhibition. Treatment of MCF-7 breast cancer cells with L-sepiapterin abrogated Tyr nitration of IκBa and increased p65 S-nitrosylation, reducing NF-κB activity. Since L-sepiapterin decreases O_2_^•−^ and increases NO amount, we can conclude that BH4 metabolism alteration modulates the role of NF-κB in cancer and probably the activity of other signaling pathways. Finally, L-sepiapterin inhibited clonogenic capability of MCF-7 and MDA-231 cells in vitro which was shown to be dependent on NO and PKG. MDA-231 xenografts tumor growth and progression were also impaired in the presence of L-sepiapterin. Although MCF-7 and MDA-231 breast carcinoma cells express both iNOS and eNOS isoforms, these results suggested that iNOS is uncoupled since the iNOS selective inhibitor 1400 W impaired the L-sepiapterin-induced cGMP levels [35].

Previously, the same group described that L-sepiapterin impaired chemically induced murine colitis and azoxymethane-induced colorectal cancer [52]. Inflammation is one of the hallmarks of colorectal cancer contributing to malignant transformation and tumor progression [71,72]. Activation of pro-inflammatory NF-κB signaling pathway inducing increased iNOS expression is one of the mechanisms of colorectal cancer development [38,73]. Besides that, the participation of eNOS isoform in colorectal cancer carcinogenesis has been shown by other authors [38,39,73,74,75]. Interestingly, Gochman et al. demonstrated increased iNOS expression and nitrotyrosine content in colitis and colorectal carcinoma, which is caused by peroxynitrite, an RNS and biomarker of oxidative damage, formed by a reaction between nitric oxide and O_2_^•−^ [76]. Since peroxynitrite formation is a very fast reaction, O_2_^•−^ and NO must be in close proximity [77], which normally occurs when both species are produced by NOS. Moreover, Youn et al. demonstrated that resveratrol, an antioxidant, abrogated superexpression of iNOS and DSS-induced colitis, indicating that iNOS is uncoupled and producing O_2_^•−^ [78]. Burhanuddin et al. also found increased iNOS expression in DSS/AMO-induced colorectal cancer, but not measured NOS activity [79]. ROS is also induced by inflammation [80], implicating NOS uncoupling in colorectal cancer development. Cardnell et al. showed that sepiapterin decreases inflammation through reduction of infiltrating neutrophils and macrophages and proinflammatory cytokine expression, which in turn impaired tumor growth. Reduction in BH4/BH2 ratio was inconclusive since there was a trend for lower BH4/BH2 after DSS treatment that was rescued by sepiapterin. On the other hand, sepiapterin reversed the increase in cGMC levels and Tyr nitration, a hallmark of peroxynitrite formation, after DSS treatment. In conclusion, these results suggested for the first time the involvement of NOS uncoupling in colitis-induced colorectal carcinoma [52].

Campos et al. demonstrated for the first time evidence of the association between NOS uncoupling and murine melanocyte malignant transformation. Melanocytes submitted to a chronic stress condition showed increased *Nos3* expression. Moreover, O_2_^•−^ was suppressed by L-NAME (N-Nitro-L-arginine methyl ester), a NOS inhibitor [81]. The same group proposed that NOS uncoupling in murine melanoma cells was a result of decreased BH4 bioavailability since L-sepiapterin treatment restored NO amount and reduced O_2_^•−^ concentration [53]. Recently, our group showed that although there is no difference between the absolute concentration of BH4 in human melanoma cells when compared to normal melanocytes, BH4/BH2 ratio was lower in tumor cells. In addition, these melanoma cells showed an increase in iNOS and eNOS protein expression. In endothelial cells, NOS uncoupling can be a result of altered NOS and BH4 stoichiometry which means that there is not enough BH4 to catalyze NO formation. Supporting this finding, melanoma cells produced higher levels of O_2_^•−^ and lower levels of NO. Treatment with BH4 increased the BH4/BH2 ratio, NO concentration, and decreased O_2_^•−^. More importantly, BH4 attenuated cell proliferation and induced apoptosis of metastatic melanoma cells, but not melanocytes, pointing to its use as a promising therapy [13]. The hypothesis of NOS/BH4 stoichiometry loss was demonstrated by eNOS suppression in metastatic melanoma cells. Decreased *Nos3* expression abrogated the increase in O_2_^•−^ and the reduction of NO. Moreover, cell growth in vitro and in vivo was attenuated (Melo et al., unpublished). Increased expression of all three isoforms has been shown in melanoma cells and tissues [13,40,41]. However, as mentioned, not all studies investigated NOS activity. This information is fundamental since NO and O_2_^•−^ modulate different signaling pathways in melanoma, provoking different tumor phenotypes. Furthermore, innumerous authors suggested that the imbalance between NO and O_2_^•−^ results in cancer growth and progression or tumor development impairment.

Recent data showed that GTPCH1/BH4/NO axis impaired hepatocellular carcinoma (HCC) development. *GCH1* is downregulated in HCC tissues and different cell lines by promoter methylation. Decreased *GCH1* expression is significantly associated with a higher Barcelona Clinic Liver Cancer stage and larger tumor size. Moreover, *GCH1* is correlated with poor clinical outcomes in HCC patients. *GCH1* silencing promotes proliferation in vitro and in vivo through inhibition of oxidative stress-induced AKT/p38 activation, indicating the involvement of uncoupled NOS in HCC carcinogenesis [54]. Although the status of NOS expression was not showed, the results implicate altered NOS activity. On the other hand, different studies showed the pro-tumoral activity of NO-derived eNOS/iNOS in HCC growth [82,83]. González et al. reported that sorafenib, a receptor tyrosine kinase, and MAPK pathway inhibitor, decreased eNOS activity and NO synthesis, attenuating proliferation and inducing apoptosis in HCC cell lines [83]. Moreover, the *NOS3* T-786C polymorphism (rs2070744) genotype, resulting in reduced activity of *NOS3* gene promoter and NO production, is associated with better clinical outcomes in patients treated with sorafenib [84,85]. Rahman et al. showed that higher *NOS2* and *PTGS-2* expression was correlated with poor prognosis in hepatitis C virus–positive HCC patients. However, NOS activity was not analyzed [86]. Sorafenib has been eligible for the standard treatment for patients with advanced unresectable HCC and the participation of uncoupled NOS was evaluated in primary operable HCC. Accordingly, NOS can have a dual role in HCC progression as was shown in other cancers [87].

On the other hand, Zhang et al. reported an oncogenic role of BH4 in breast cancer development. Increased sepiapterin reductase protein (SR) was found in breast cancer tissues when compared to the adjacent non-tumorigenic tissue and correlated with tumor aggressiveness. SR downregulation inhibited metastatic breast cancer cell proliferation and induced ROS-mediated apoptosis. Although NO amount was not evaluated, decreased BH4 concentration was reported in MDA-MB-231 and MDA-MB-468 breast cancer cells, suggesting the involvement of uncoupled NOS in metastatic breast cancer survival impairment [55]. The reduction of sepiapterin reductase can affect another pathway, since its inhibition impairs ornithine decarboxylase activity and decrease polyamines concentration in neuroblastomas, attenuating tumor growth [88,89]. The shift in arginine metabolism leading to high levels of polyamines will reduce NO synthesis affecting NOS function [90]. Therefore, although SR and GTPCH1 are on the same metabolic pathway, their contributions to breast cancer progression may not be the same.

In glioblastoma (GBM), *in silico* analyses showed that increased *GCH1* expression was correlated with higher glioma grade, recurrence, and worse survival. Furthermore, *GCH1* overexpression and BH4 levels elevation in GBM cells increase proliferation and decrease survival in an intracranial GBM-mouse model, which is correlated with brain tumor-initiating cells maintenance and ROS suppression [56]. GBM development is associated with high ROS levels; however, exceeded production can induce cell death [91,92]. On the other hand, increased NOS expression and NO is correlated with glioma cell proliferation [41,56,93]. Therefore, GTPCH1/BH4/NO signaling activation in GBM cells may represent a chemoresistance mechanism, preventing cell death as in melanocyte and keratinocyte cells submitted to radiation-induced ROS [51].

Increased expression of iNOS and eNOS have been related to esophageal squamous cell carcinoma (ESCC). However, neither the regulation of NOS by BH4 nor the mechanism of contribution to the pathogenesis of ESCC have not been investigated. AU-rich element RNA-binding factor 1 (AUF1) is a family of proteins involved in the post-transcriptional regulation process of mRNA and has been related as a promoter or inhibitor of cancer progression [57,94,95,96]. Gao and colleagues found that AUF1 expression is higher in ESCC when compared to normal or tumor-adjacent tissues. Interestingly, the AUF1 silencing reduced *GCH1* expression and NO amount, which in turn decreased cell growth and increased apoptosis in squamous carcinoma Eca-109 cells [57]. Although not explored by the authors, the data indicate that AUF1 downregulation causes NOS uncoupling via GTPCH1 regulation in ESCC cells. Since ESCC growth suppression was triggered by endoplasmic reticulum stress activation and increased ROS levels [97,98], NOS uncoupling can be associated with oxidative stress-induced cell death. However, to confirm this hypothesis BH4, BH4/BH2, and O_2_^•−^ analyses are required.

In a very elegant approach, Soula et al. showed that BH4 abrogates lipid peroxidation–induced ferroptosis caused by glutathione peroxidase 4 (GPX4) inhibition [58]. Using metabolism-focused CRISPR-Cas9 genetic screens it was shown that decreased expression of enzymes from the *de novo* BH4 synthesis pathway is associated with cell death triggered by RSL3, a GPX4 inhibitor, in different cancer cell lines. Exogenous BH4 supplementation increased proliferation and restored the resistance of T- and B-cell acute lymphoblastic leukemia and lymphomas to GPX4 inhibitors, indicating BH4 synthesis as a new target therapy in those tumors. The authors showed that ferroptosis impairment was caused by the ability of BH4 to act as a radical-trapping antioxidant in lipid membranes, avoiding lipid peroxidation in accordance with another study using fibroblasts [43]. It was also suggested that the protective role of BH4 was by an eNOS-independent mechanism. However, it was assumed that NO formation by NOS promotes the oxidation of BH4 to BH2, reducing BH4 bioavailability, which in turn triggers NOS uncoupling and O_2_^•−^ generation. BH4 is not consumed in the reaction catalyzed by NOS, instead, it is regenerated by the enzyme itself, not resulting in a BH4 decrease [99]. Moreover, BH4, NO, and O_2_^•−^ levels were not evaluated to be able to infer about NOS activity. NOS inhibition or eNOS silencing renders leukemic cells more sensitive to RSL3, suggesting the protective function of NO. This data is in agreement with other studies showing that NOS activity is important in leukemia cell survival [41,55].

In the early stages of colorectal cancers, PTPS was found to be elevated. Under hypoxia, a common characteristic of aggressive cancers, AMPK-induced PTPS phosphorylation, which in turn promotes LTBP (Latent TGF-β binding protein)-PTPS-iNOS interaction leading to LTBP nitrosylation and ubiquitin-dependent degradation. In this condition, TGF-β secretion is increased contributing to colorectal tumor growth. *PTS* silencing impaired LTBP nitrosylation and cell proliferation, showing the involvement of the BH4/NO pathway in colorectal cancer progression [59]. The apparent conflict with the study from Cardnell et al. can be explained by the fact that the authors used an AMS/DSS-induced murine colorectal cancer model. The treatment with AMS/DSS induces oxidative stress, causing BH4 decrease and consequently, NOS uncoupling [52], contributing to malignant transformation. In this case, L-sepiapterin supplementation decreased tumor incidence, but not tumor growth, suggesting the role of uncoupled NOS in colorectal malignant transformation instead of cancer progression.

### 4.2. Tumor Microenvironment and Angiogenesis

Heterogeneous cell types, including fibroblasts, endothelial, and immune cells, along with extracellular matrix components comprise the tumor microenvironment (TME). Through the secretion of various factors, cancer cells can reprogram and control the function of the surrounding environment, contributing to biological processes, such as angiogenesis [100].

Angiogenesis is a physiological process consisting of the growth of new blood vessels from pre-existing ones, providing oxygen and nutrient supplies. In cancer, pathological angiogenesis contributes to tumor development, increasing proliferation, growth, and metastasis [1,101]. Angiogenesis is maintained by the overproduction of proangiogenic factors, including growth factors, matrix proteinases, and extracellular matrix molecules [100,102]. Different approaches have already shown that eNOS-derived NO signaling in endothelial cells is a critical mechanism of vascular homeostasis, mediating angiogenesis and contributing to increased cell proliferation and migration of endothelial cells and consequently to tumor progression [103,104,105]. Since eNOS activity requires BH4 as a cofactor for NO production, it was shown that the maintenance of its bioavailability is essential for angiogenesis by many groups using different technical procedures [60,61,106,107].

Experiments in vitro demonstrated that increased *GCHI* expression and L-sepiapterin or BH4 supplementation augmented NO production, cell proliferation, migration, and the capacity of endothelial cells of different species to form capillary-like structures through PI3K/AKT signaling pathway activation [61,106,108]. Chen et al. showed that L-sepiapterin triggers GTP-bound wild-type Ras, increasing AKT^Ser473^ and eNOS^Sei1177^ phosphorylation and NO production. Increased cell proliferation, migration, and tube formation required PI3K signaling since LY treatment, a PI3K signaling inhibitor, abrogated it. Furthermore, Ras activation and PI3K/Akt/eNOS up-regulation were impaired by L-NAME, suggesting a positive feedback mechanism. It was shown that NO caused S-nitrosylation (SNO) of p21Ras in Cys118, triggering the activation of ERK/MAPK signaling pathway and the proliferation of innumerous cells as neural stem cells, breast cancer, and endothelial cells [109,110,111]. Accordingly, AKT activates eNOS, increasing NO synthesis that promotes S-nitrosylation of Ras in Cys118, which drives the PI3K/Akt pathway to maintain angiogenesis and consequently growth of tumors.

On the other hand, the treatment with 2,4-diamino-6-hydroxypyrimidine (DAHP), a GTPCHI inhibitor, and decreased *GCHI* expression in vitro impaired angiogenesis through abrogation of AKT activation, eNOS phosphorylation, NO production, and consequently reduction of endothelial cell proliferation, migration, and tubulogenesis [60,62,112]. The treatment of endothelial cells with ribavirin, an IMP dehydrogenase inhibitor, decreased GMP synthesis, the GTP cyclohydrolase substrate, causing BH4 and consequently NO reduction, which in turn impaired cell proliferation and tube formation [106].

Many authors evaluate the participation of BH4 in angiogenesis in vivo. To analyze the proangiogenic potential of BH4 in tumor stromal fibroblasts, BALB/c SCID mice were implanted with a construction where the gene *GCH1* was cloned into a plasmid that contains tetracycline responsive element under cytomegalovirus promoter control. In the presence of doxycycline (DOX), *GCH1* expression is abrogated. Mice with tumors around 100 mm^3^ in size (25th day after implantation) were then fed with DOX in drinking water to inhibit *GCH1* expression or injected with DAHP (300 mg/kg/day) for 7 days. Although angiogenesis was reduced, as showed by decreased CD34-positive microvessels, tumor growth was not inhibited [61]. Interestingly, eNOS expression was also reduced in DOX- or DAHP-treated mice. However, the BH4 concentration was not reduced in DAHP-treated animals, only after the treatment with DOX, suggesting that angiogenesis reduction was BH4-independent. DAHP may have additional inhibitory effects on tumor angiogenesis through downregulating of eNOS expression. Furthermore, it was shown that DAHP reduced the expression of cytokine-induced expression of vascular cell adhesion molecule 1 (VCAM-1) and the transcription factor NF-κB in endothelial cells, both proteins involved in the angiogenic process [113,114,115,116,117]. Therefore, the decreased neovascularization observed after DAHP treatment can be associated with the inhibition of other angiogenic factors.

On the other hand, Dai et al. observed that DAHP treatment (80 mg/kg/day) for two weeks once tumors reached 100 mm^3^ in size inhibited AKT/eNOS pathway activation and decreased BH4 and NO concentration in hepatocellular carcinoma tissue. Hence, CD31 staining was significantly lower in DAHP-treated mice showing impairment of angiogenesis and tumor growth [60]. Some factors can be associated with these different results between the two studies. Although they observed reduced angiogenesis in DAHP-treated animals, only the treatment with 80 mg/kg/day for two weeks abrogated tumor growth, suggesting that this phenomenon is dose and time-dependent. Moreover, the animals used by Dai et al. were male mice aged 4–6 weeks, while Chen et al. worked with 6- to 8-week-old females [61]. Finally, and more importantly, Chen et al. did not observe a decrease in BH4 content in tumor stromal fibroblasts after DAHP treatment. However, it was reported that the growth of cancer-associated fibroblast (CAFs) is BH4-dependent [63].

CAFs are found in the tumor microenvironmental surrounding the malignant lesion and, through dysregulated cell signaling and communication with tumor cells, contribute to malignant transformation, proliferation, metabolic program, and metastasis [118]. Furthermore, the presence of CAFs has been correlated with poor outcomes in different cancers, including breast cancer [119,120,121]. GTPCH1 was overexpressed in stromal fibroblasts of breast cancer when compared with normal tissue. Moreover, high GTPCH1 was significantly associated with aggressiveness and low recurrence-free survival [63]. Interestingly, GTPCH1-expressing fibroblasts induced BH4-dependent breast cancer proliferation and migration by secreting angiopoietin-1 (Ang-1), which in turn triggers Tie2 phosphorylation and the activation of Ras/PI3K/AKT and ERK pathways. Furthermore, breast tumor growth in vivo and angiogenesis was also enhanced by GTPCH-expressing fibroblast complaints [63]. The authors suggested that Ang1/Tie2 interaction increases breast cancer growth through activation of Ras/PI3K/Akt signaling pathway as was shown in endothelial cells [61]. This study did not investigate NO/O_2_^•−^ amount or NOS expression; therefore, the relationship between BH4 and enzyme activity was not discussed. However, studies have indicated that NO can induce Ang1/Tie2 interaction [122,123]. Zacharek et al. showed that treatment with a NO donor, (Z)-1-[N-(2-aminoethyl)-N-(2-ammonioethyl) aminio] diazen-1-ium-1,2-diolate (DETA-NONOate), increased Ang1 and Tie2 expression in stroke rats. Furthermore, DETA-NONOate induced capillary tube formation in mouse brain endothelial cells, attenuated by Ang1 blockage [123]. Since NO-derived NOS also stimulates endothelial cell proliferation by Ras/PI3K/AKT activation and NO is associated with breast cancer growth [124,125], BH4-induced NO can contribute to breast cancer progression. Moreover, eNOS activity is also increased by Ang1/Tie2-induced Ras/PI3K/AKT activation, suggesting a feedback mechanism [126]. Therefore, further studies are needed to investigate whether Ang1 induction is related to NO production by NOS coupling or directly by increased BH4 in CAFs.

Showing that the anti-angiogenic and consequently anti-tumor role of DAHP is time-dependent, Pickert et al. showed that the administration of DAHP (100 mg/kg) twice daily orally immediately after tumor cells inoculation reduced the formation of blood vessels as indicated by decreased CD31 and αvβ3 integrin staining [62]. Importantly, DAHP treatment diminished BH4 plasma concentration. Reduction of angiogenesis attenuated tumor growth of B16F10 murine melanoma cells and HT29 human colon cancer cells in vivo. Blood vessels formation in shGCH1-transduced HT29 xenotransplants was analyzed to investigate if the production and release of BH4 from cancer cells also contribute to angiogenesis. In addition to decreasing CD31 and αvβ3 integrin amount, the development of shGCH1-transduced HT29 xenotransplants was reduced when compared with control cells. Moreover, DAHP treatment has been associated with impaired immune-induced tumorigenesis, since tumor-associated macrophages (TAMs) with an antitumoral M1-*like* phenotype were infiltrating tumor microenvironmental. These data are in accordance with the studies showing that macrophages polarized towards an anti-inflammatory M2 phenotype instead of M1-like phenotype are involved in high angiogenic activity [127,128]. However, the mechanism underlying increased angiogenesis by pro-tumoral M2 polarized macrophages is not associated with increased NOS expression but instead with elevated arginase activity and consequently with reduced NO concentration [129]. Therefore, the axis GTPCH1/BH4/NOS/NO could be a compensatory pathway to maintain NO-induced angiogenesis.

Important components of the TME in various types of cancer, TAMs exhibit a wide spectrum of phenotypic and functional profiles regulated by different signals of the surrounding environment [130,131]. Therefore, functional reprogramming of TAMs to a more anti-tumorigenic profile has been explored as presenting a therapeutic potential [132,133]. CA1d human breast cancer cells and M2 macrophages treated with sepiapterin produced higher levels of NO than polyamines. This rise in NO/polyamine ratio was accompanied by p-STAT3 inhibition and programmed death-ligand 1 (PD-L1) downregulation in mammary tumor cells. Furthermore, sepiapterin treatment of M2-macrophages increased iNOS, STAT1, and IL-12 (M1 macrophage markers) expression, while decreasing M2 markers (CD163, IL-10, and STAT3) [64]. NO and polyamines are products of arginine metabolism by NOS and arginase, respectively. Studies have reported that beyond substrate competition, these two pathways can inhibit each other [134,135]. Furthermore, both have an important role in macrophage polarization, so that NOS/NO is related to M1; on the other hand, arginase/polyamine to the M2 pro-tumor phenotype [129]. In fact, elevated levels of polyamines have been related to different types of cancer and associated with a poor prognosis of breast cancers [136,137]. Interestingly, ex vivo analysis showed that 100 μM sepiapterin reduced PD-L1 expression and tumor epithelial density (cytokeratin 14 staining), while reprogramming TAMs from M2 to M1 phenotype in culture tumors derived from MMTV-PyMT mice [64]. PD-L1 expression has not only been associated with poor prognosis in breast cancer patients but was also considered a target for immunotherapy [138,139,140], with an important role of TAMs in this scenario [141]. Therefore, redirecting arginine metabolism by BH4 synthesis in cancer cells and TAMs can improve breast cancer immunogenicity.

Recently, the effects of BH4 on T cells and its role in breast cancer were also investigated. Although the normal development of T cells in mice knocked to *Gch1* specifically for these cells, there was a reduction in BH4 levels accompanied by a decrease in the proliferation of mature T cells induced via TCR (T cell receptors). A similar result was shown by inhibiting sepiapterin reductase through the SPRi3 inhibitor. On the other hand, the increased BH4 levels by *GCH1* overexpression or L-sep/BH4 treatment enhanced the proliferation of stimulated CD4^+^ and CD8^+^ T cells. Interestingly, on the orthotopic breast cancer model (E0771 cells), the tumor growth was rejected in *GCH1*-overexpressing mice. Consistently, BH4 supplementation (100 mg/kg^−1^/day, intraperitoneally) decreased tumor growth and increased tumor-infiltrating CD4^+^ and CD8^+^ T cells. Furthermore, stimulated BH4-deficient T cells showed decreased iron levels and reduced mitochondrial respiration and oxygen consumption [65]. Thus, the authors indicate mitochondrial dysfunction via defective iron-redox cycling of cytochrome *c* as a mechanism for the effects of BH4 deficiency in TCR-activated T cells. However, the antioxidant activity of BH4 is controversial [19] and its role as a cofactor of NOS cannot be discarded. Although there is no difference in iNOS expression in stimulated T cells, other NOS isoforms could be involved, since its activity has been described in these cells [142,143]. In addition, the fact that *Gch1*-ablated T cells do not show a difference in nitrite levels compared to control cells does not completely exclude the participation of NOS, since the enzyme in stimulated T cells may be in a state of partial uncoupling, that is, producing NO and O_2_^•−^ [144]. Consistently, increased O_2_^•−^ levels were found in stimulated T cells, which were potentiated in BH4-deficient cells [65].

The fact that hypoxia induces GTPCH1 expression and BH4 synthesis corroborate their critical role in angiogenesis [62]. Reduced oxygen apport triggers the expression of the transcription factor hypoxia-inducible transcription factor (HIF-α), which in turn induces the expression of more than 60 genes, including VEGF that induces NO signaling-mediated angiogenesis [145].

Angiogenic vasculature structure found in solid tumors is poorly organized, leading to heterogeneous blood flow, increased interstitial pressure, and intermittent hypoxia contributing to radioresistance and the low ratio of drug delivery [146]. One of the mechanisms associated with dysfunctional tumor vasculature is NOS uncoupling caused by the decreased bioavailability of BH4 [147]. Normalization of abnormal cancer vasculature can result in a transient period to ameliorate drug delivery [125,148]. In a spontaneous breast tumor mice model, L-sepiapterin induced NOS recoupling, which in turn reduced hypoxia, restored perfusion leading to increased doxycycline uptake and cell death. Moreover, sepiapterin also increased radiation-induced apoptosis [66]. Although NO-derived NOS improves tubulogenesis through stimulation of endothelial cell proliferation and migration, uncoupling NOS also contributes to disruption of angiogenic vasculature impairing treatment. It is important to note that there is a therapeutic window where sepiapterin supplementation can be used to abrogate cancer cell resistance to radio- and chemotherapy.

### 4.3. Migration and Invasion

Metastasis is a complex process comprising a sequence of steps that begins with local invasion (migration and extracellular matrix degradation), then intravasation into lymphatic and blood vessels, survival in hematogenous and lymphatic systems, extravasation into the parenchyma of distant tissues, and finally the formation and growth of small niches of cells, the metastatic lesions [149,150].

Kanugula et al. showed that statin treatment reduced invasion and induced apoptosis of triple-negative breast cancer cells, by iNOS-mediated NO production [67]. Statins are inhibitors of 3-hydroxy-3-methylglutaryl coenzyme A (HMG-CoA) reductase (a rate-limiting enzyme of the mevalonate pathway) and have been used in the treatment of patients with cardiovascular diseases [151,152]. Fluvastatin enhanced iNOS expression, NO production, and nitrite levels in breast cancer cells, while reduced transferrin receptor (Tfr1) expression and iron uptake leading to cell death. These effects were abrogated in the presence of mevalonate, ADMA, or 1400 W. In addition, this statin decreased H_2_O_2_ levels and downregulated MMP-2 and MMP-9 transcripts, inhibiting the invasive potential of the cells, which was reversed in the presence of a catalase inhibitor [67]. Since studies reported that fluvastatin and others statins increased eNOS-mediated NO production via upregulation of GTPCH and consequent increase BH4 levels [153,154,155], the antitumor effects of fluvastatin found by Kanagula and colleagues can be associated with iNOS coupling. Consistently, the authors showed that sepiapterin treatment (50 μM for 24 h) was able to reduce Tfr1 expression and iron uptake in MDA-MB-231 cells. Furthermore, inhibition of iNOS by 1400 W reversed H_2_O_2_ levels reduction and the anti-invasive effect of fluvastatin in these cells [67]. Although not exploring the role of BH4/NOS, other studies have also demonstrated the anti-tumor propriety of fluvastatin on breast, hepatocellular, ovarian, and prostate cancer cells [156,157,158].

In ovarian cancer cells, sepiapterin abrogated the increase in migration and proliferation triggered by vascular endothelial growth factor-A (VEGF-A) and p70S6K-dependent VEGRF2 expression in a NO-independent mechanism. Surprisingly, sepiapterin increased migration and proliferation of ovarian cancer cells in the absence of growth factors, which is mediated by a NO-dependent activation of ERK, AKT, and p70S6K signaling [68]. Since VGFR2 activation promotes ROS increase which is associated with ovarian cancer development [159,160,161], impairment of cell growth in the presence of sepiapterin can be attributed to NOS recoupling. These results suggest that the role of sepiapterin in cancer is related to the modulation of signaling pathways by NO or ROS. Elevated expression of the three NOS isoforms has been associated with ovarian cancer carcinogenesis [40,162]. Some authors documented that increased iNOS expression is correlated with poor prognosis. In iNOS-positive patients, the authors observed an increase in the risk of disease relapse, lower disease-free survival period, and high death incidence [40,163,164]. However, NOS activity was not evaluated. So, expression of the NOS isoforms can be useful as a prognostic factor in ovarian cancer; however, as a therapeutic target, its real activity must be taken into account. It is also important to note, that NOS uncoupling can be partial, implicating the NO and O_2_^•−^ synthesis at the same time.

Some important acquisitions of cancer cells during tumor progression and metastatic colonization are the ability of anchorage-independent growth and resistance to a programmed cell death known as *anoikis*. This type of apoptosis is induced by loss of attachment to extracellular matrix or inappropriate cell-matrix interactions, preventing cell adhesion to inadequate locations [165,166]. Campos et al. also showed that uncoupled eNOS contributes to *anoikis*-resistance of melanocytes submitted to anchorage impediment [81]. Superoxide production was higher in melanocytes maintained in suspension, which was reversed in the presence of L-sep or inhibiting NOS by L-NAME. Curiously, DAHP treatment enhanced O_2_^•−^ and reduced NO levels in these cells. Furthermore, sepiapterin and Mn(III)TBAP (a superoxide scavenger) were able to decrease melanocyte and metastatic melanoma cell survival in suspension, but not in adhesion [53]. In fact, malignant melanocyte transformation has been associated with higher production of ROS, characterizing disrupted redox homeostasis [167]. The control in ROS and NO concentrations can mediate the acquisition of the appropriate phenotype in different steps of tumor progression and prevents cancer cell death under adverse conditions [168]. Cell lines corresponding to distinct stages of melanoma progression increased O_2_^•−^ production and decreased NO levels compared to parental melanocytes. In addition, in vivo analysis showed that the treatment with L-NAME before and during sequential cycles of anchorage blockade impairs melanocyte malignant transformation [53]. Taken together, these results indicate that BH4 synthesis and eNOS coupling has anti-tumor effects on melanoma development.

## 5. Conclusions

**Figure 3:**
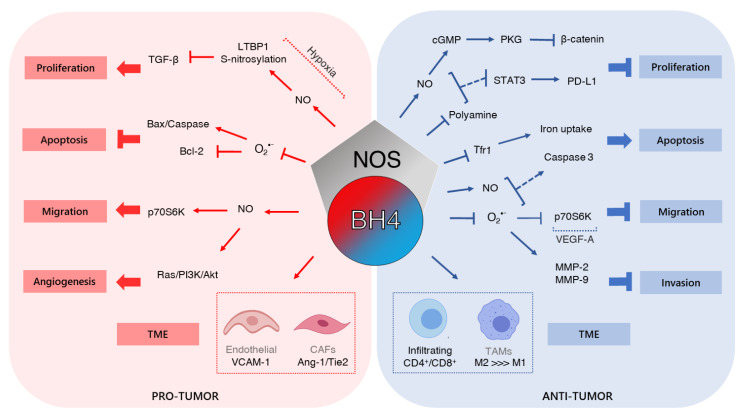
Schematic presentation of the dual mechanisms of the BH4/NOS axis in acquired capabilities along with cancer progression. BH4 pro-tumor activity comprises proliferation induction through TGF-β pathway activation, apoptosis abrogation by Bax inhibition and Bcl-2 increase, p70S6K-dependent promotion of migration, and angiogenesis stimulation by Ras/PI3K/Akt signaling. As anti-tumor, BH4/NOS axis induces apoptosis due to caspase-3 activation and iron uptake decrease, reduces proliferation by decreasing polyamine concentration, and impairs migration and invasion by reducing VEGF-A/p70S6K pathway and MMP-2 and MMP-9 activity, respectively.

Altered signaling pathways are associated with malignant transformation and the acquisition of an aggressiveness phenotype. The expression, activity, and cell localization of these transduction signals are regulated by innumerous factors, including ROS and NO-induced post-translational modifications. Different studies have shown that ROS and NO amount and the balance between these reactive species contribute to tumor growth, chemoresistance, and metastasis. Moreover, the contribution of ROS and RNS from tumor microenvironmental in cancer development has to be considered. Since NOS can be a source of both ROS and NO, understanding its activity is essential to improve cancer management. Therefore, the dual role of the BH4/NOS pathway in carcinogenesis can be explained by the status of transduction pathways found in distinct cancers (Figure 3).

## Figures and Tables

**Table 1:** Studies showing the role of BH4 and NOS activity in the progression of different types of cancers.

Cancer Type	Altered Tumor Capability	NOS Isoforms	Management Methods of BH4 Levels	BH4/NOS Role	Reference
Melanoma	Growth/Apoptosis in vitro	eNOS, iNOS	BH4/L-sep supplementation	Anti-tumor	[13]
Breast	Growth in vitro/in vivo	eNOS, iNOS	L-sep supplementation	Anti-tumor	[35]
Colorectal	Growth in vivo	NOS	L-sep supplementation	Anti-tumor	[52]
Melanoma	Anoikis in vitro, Growth in vivo	eNOS	L-sep/DAHP supplementation	Anti-tumor	[53]
HCC	Growth in vitro/in vivo	NOS	*GCH1* silencing BH4 supplementation	Anti-tumor	[54]
Breast	Growth/Apoptosis in vitro	-	*SPR* silencing	Pro-tumor	[55]
Glioblastoma	Growth in vitro/in vivo	-	*GCH1* overexpression *GCH1* silencing	Pro-tumor	[56]
ESCC	Growth/Apoptosis in vitro	-	*GCH1* silencing *GCH1* regulation	Pro-tumor	[57]
Leukemia, lymphoma	Growth in vitro	eNOS	*GCH1/SPR/PTS* knockout BH2 supplementation	Pro-tumor	[58]
Colorectal	Growth in vitro/in vivo	iNOS	BH4 supplementation *PTPS* silencing	Pro-tumor	[59]
HCC	Angiogenesis/Growth in vivo	eNOS	DAHP supplementation	Pro-tumor	[60]
CAFs	Growth/Angiogenesis in vivo	eNOS	DAHP supplementation *GCH1* silencing	Pro-tumor	[61]
Colon, Breast, melanoma, TAMs	Angiogenesis/Growth in vivo/in vivo	eNOS	DAHP supplementation, *GCH1* silencing	Pro-tumor	[62]
Breast, CAFs	Growth in vitro/in vivo Angiogenesis in vivo	-	DAHP supplementation *GCH1* silencing	Pro-tumor	[63]
Breast, TAMs	Growth ex vivo	eNOS, iNOS	L-sep supplementation	Anti-tumor	[64]
Breast, T cells	Growth in vivo	iNOS	BH4/L-sep supplementation *GCH1* overexpression/silencing	Anti-tumor	[65]
Breast	Angiogenesis/Apoptosis in vivo	NOS	L-sep supplementation	Anti-tumor	[66]
Breast	Invasion/Apoptosis in vitro	iNOS	L-sep supplementation	Anti-tumor	[67]
Ovarian	Growth/migration in vitro	NOS	L-sep supplementation	Anti/pro- tumor	[68]

BH4: Tetrahydrobiopterin; NOS: nitric oxide synthase; iNOS: inducible nitric oxide synthase protein; eNOS: endothelial nitric oxide synthase protein; DAHP: 2,4-diamino-6-hydroxypyrimidine; L-sep: L-sepiapterin; BH2: dihydrobiopterin; *GCH1*: cyclohydrolase gene; PTPS: 6-pyruvoyltetrahydrobiopterin synthase protein; *SPR*: sepiapterin reductase gene; TAMs: tumor-associated macrophages; CAFs: cancer-associated fibroblast; ESCC: esophageal squamous cell carcinoma; HCC: hepatocellular carcinoma.
